# A Wife Saving Experiment

**Published:** 1856-02

**Authors:** 


### A WIFE SAVING EXPERIMENT.

It boots me little what men think of me—WHAT I AM is the Polar star on which I fix my gaze as I toss on life's billowy ocean—it is the foundation stone on which I stand, and feel myself more impregnable than Gibraltar's rock; a fountain of satisfaction to me welling up unceasingly, as pure as the dew-drops and sweeter than honey from the rose-bud. That is, speaking of wives and the way to manage them!! We know what kind of a one we have, and can afford the surmisings of others, just as Longworth, the millionaire of the West, whose tax bill alone, exceeds thirty thousand dollars a year, can afford, as he does, to dress almost as well as a common dray-man. Well, we are wool gathering to-day, but what we began this article to say is, if you find your wife out of sorts, cross, hard to please, about half-way between sick and well, do not attempt to buy her over with a new dress, that is like inhalation for consumption, a mere diversion while the money is being abstracted from your purse, or like a pill of opium, alleviating only, eradicating nothing; or like cutting off a cancer, to bre[illegible] out in a worse form at some other place, but take her to the country, some ten or twenty miles distant, keep her going all the time, and in eight or ten days return home; you will find her in all desirable respects a different woman,—it is an almost inevitable result arising from change of air, change of food, change of association, and not least from that *expansion of view* which attends larger associations. This is an important item in travel, one of its greatest benefits. The most celebrated travellers are the men who are most liberal in their feelings, the least dogmatical, the least impatient of opinions contrary to their own. By indulging your wives in frequent excursions, three or four times in a year, you will enlarge their views of things, increase their sociabilities, improve their health and their tempers, and more, you will find they have an increasing love for home.

